# Obesity, Metabolic Syndrome, and Adipocytes

**DOI:** 10.1155/2011/721686

**Published:** 2011-07-24

**Authors:** M. V. Dodson, P. S. Mir, G. J. Hausman, L. L. Guan, Min Du, Z. Jiang, M. E. Fernyhough, W. G. Bergen

**Affiliations:** ^1^Department of Animal Sciences, Washington State University, Pullman, WA 99164, USA; ^2^Agriculture and Agri-Food Canada Research Centre, Lethbridge, CA, Canada T1J 4B1; ^3^USDA-ARS, Richard B. Russell Agricultural Research Station, Athens, GA 30604, USA; ^4^Department of Agricultural, Food and Nutritional Science, University of Alberta, Edmonton, AB, Canada T6G 2P5; ^5^The Hartz Mountain Corporation, 400 Plaza Drive, Secaucus, NJ 07094, USA; ^6^Program in Cellular and Molecular Biosciences/Animal Sciences, Auburn University, Auburn, AL 36849, USA

## Abstract

Obesity and metabolic syndromes are examples whereby excess energy consumption and energy flux disruptions are causative agents of increased fatness. Because other, as yet elucidated, cellular factors may be involved and because potential treatments of these metabolic problems involve systemic agents that are not adipose depot-specific in their actions, should we be thinking of adipose depot-specific (cellular) treatments for these problems? For sure, whether treating obesity or metabolic syndrome, the characteristics of all adipose depot-specific adipocytes and stromal vascular cells should be considered. The focus of this paper is to begin to align metabolic dysfunctions with specific characteristics of adipocytes.

## 1. Introduction

**Figure 1:**
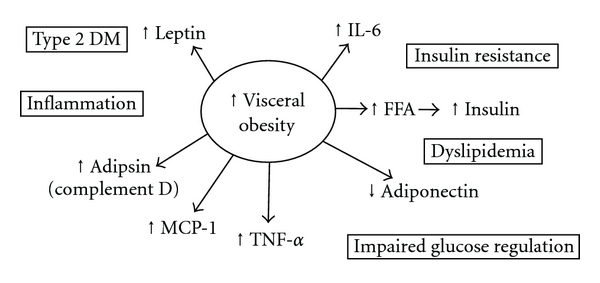
Potential causal agents in visceral obesity and the metabolic syndrome. The most dramatic form of obesity is characterized by excess visceral adipose tissue, which has been shown to be related to progression of symptoms of metabolic syndrome [11]. Among the symptoms of this syndrome is insulin resistance, which appears to be associated with increases in concentrations of inflammation markers in blood. Morphologically in lipid engorged adipocytes, the nucleus and the lipid synthetic apparatus of cells is marginalized and may negatively affect further fat synthesis leading to hyperglycemia or hypercholesterolemia which is commonly observed in individuals with metabolic syndrome. Individual regulatory agents shown have been recently described [12]. DM: diabetes mellitus; FFA: free fatty acid; MCP-1: monocyte chemo attractant protein-1; TNF-*α*: tumor necrosis factor alpha; IL-6: interleukin 6.

Our modern lifestyle has led to the diminished use of consumed energy and to an increasing population of obese individuals. Obesity is costly [1, 2], can result in a poor quality life [2, 3], and causes an early death [1, 2]. Metabolic syndrome is manifested by many symptoms like elevated intramyocellular lipid, intramuscular lipid, blood insulin, glucose, cholesterol, triacylglycerol, increases in blood pressure, risk of cardiovascular disease, and heightened chances of being diagnosed with type 2 diabetes. The progression of both obesity and metabolic syndrome is reaching epidemic proportions [4–9] and appears to occur at increasingly earlier ages [10]. A general dietary, or metabolic, approach to combat both obesity and metabolic syndrome has had only limited success, but both are generally linked to visceral fat and a host of interacting physiological and pathological processes (Figure 1, Table 1).

## 2. Adipose Tissue as a Sink for Excessive Fatty Acids

White adipose tissue is critical for the deposition of excessive fatty acids. Surgical implantation of adipose cells into transgenic, adipose-devoid mice reinstated insulin sensitivity, decreased plasma glucose levels, and normalized insulin concentrations [13]. Adipocyte deficiency in human subjects with congenital generalized lipodystrophy is typically accompanied by severe insulin resistance, hyperinsulinemia, hyperglycemia, hypertriglyceridemia, and fatty livers [14]. It has been shown that the efficiency of adipose tissues acting as a repository for excess energy is reduced in transgenic mice which express high levels of preadipose factor-1, resulting in decreased adipose tissue and induction of glucose intolerance and hypertriglyceridemia [15]. Transgenic mice, with 99% less white adipose tissue and inactive brown adipose tissue, developed insulin resistance manifested by threefold elevated plasma glucose levels and 50- to 400-fold elevated insulin levels [16, 17]. White adipose tissue-specific proteins, including perilipin and fat-specific protein of 27 kDa (FSP27), promote sequestration of triglycerides in adipose tissue, which reduces circulatory fatty acid concentration, improving insulin sensitivity in peripheral tissues [18]. FSP27 mediates the formation of unilocular lipid droplet in adipocytes and its knockout enhances lipolysis, leading to insulin resistance [19]. 

Adipose tissue development is characterized by both adipocyte hyperplasia and hypertrophy. Limiting adipocyte hyperplasia leads to accumulation of lipids in existing adipocytes, resulting in hypertrophy. The proteins downstream of tyrosine kinases-1 (Dok1) are important for adipogenic differentiation and adipocyte hyperplasia. Dok1 activates p120 Ras GTPase-activating proteins, which inhibit the proliferation of preadipocytes and promote their differentiation and lipid accumulation, resulting in hypertrophy of existing adipocytes [20]. It is known that lipid-laden adipocytes are correlated with insulin resistance [21].

In summary, adipose tissue functions as a sink for excessive fatty acids, and the impairment of fatty acid storage function of adipose tissue results in the deposition of fatty acids in other tissues, such as key insulin responsive tissues, muscle and liver, leading to insulin resistance. If metabolic syndromes (specifically) are undiagnosed and allowed to progress, patients will experience persistent hyperinsulinemia, resulting in detectable apoptosis of pancreatic *β*-cells and Type II diabetes [22].

## 3. Adipose Depots and Metabolic Syndromes

While visceral adipose tissue appears primarily involved in metabolic syndromes, there are three other major adipose depots that also may have a role in the etiology of obesity and/or metabolic syndrome, including visceral, subcutaneous, intermuscular, and intramuscular (including intramyocellular lipid) depots. Not all adipose depots possess the same physiology, and the distribution of adipocytes within the four major adipose depots plays a role in the severity of obesity and ultimately metabolic syndromes. This is probably due to the inherently different responses by the collective populations of different types of adipocytes in the distinct adipose depots. 

Obtaining knowledge of how cell populations in depot-specific adipose tissue manage the dynamics of energy partitioning is of growing importance [23, 24]. However, this is a difficult area of physiology to grasp as not only are the adipose depots different, but so are the adipocytes within any one adipose depot [12]. Moreover, because stromal vascular cells, preadipocytes, and mature adipocytes are sources of proliferative-competent progeny cells in adipose tissue, the traditional descriptions of adipogenesis and differentiation no longer hold true [12]. This implies that novel experimental designs must be employed in order to address depot-specific adipocyte cellularity during development, growth, and metabolism. To confound matters further, the adipose tissue stromal vascular content is also depot dependent and may, in part, dictate the potential of disease severity. Firstly, these cells may provide preadipocytes for increasing the number of lipid-assimilation cells in the depot. Also, as the extent of vascularity and connective tissue composition and deposition may be under intrinsic control different from the adipocyte itself, stromal vascular cells may become a key player in adipocyte physiology. For example, connective tissue deposition and organization are decreased in depots with a higher proportion of larger adipocytes (internal or visceral depots). Impaired adipose tissue blood flow associated with severe adipocyte hypertrophy in these depots may induce hypoxic conditions which, in turn, may destabilize the adipocyte extracellular matrix (ECM) resulting in a number of adverse conditions. Also, severe adipocyte hypertrophy *per se* may adversely influence both the capillary and adipocyte ECM stability. All components of adipose tissue depots must be considered as being potentially involved in adipose tissue-related disease. 

Increasing energy utilization via exercise or weight loss provides a transient opportunity for energy storage in existing adipose cells, improves insulin sensitivity, and allows consumed, but as yet unused glucose, to be stored as lipid. However, if exercise/weight loss is inefficient, what becomes of the unutilized glucose? Moreover, is the manifestation of type II diabetes or hypercholesterolemia an indication of where feedback inhibition of depot-specific cellular metabolic processes exists? Also, is the hypercholesterolemia seen in metabolic syndrome caused by the inability of the body to synthesize lipid from acetate? Moreover, is the manifestation of type II diabetes or hypercholesterolemia an indication of where feedback inhibition of depot-specific cellular metabolic processes exists? Careful scrutiny of adipocytes may well address these questions and will provide knowledge about specific populations of adipocytes in the development of obesity/metabolic syndromes.

## 4. Targets with Which to Combat Obesity and Metabolic Syndromes

**Figure 2:**
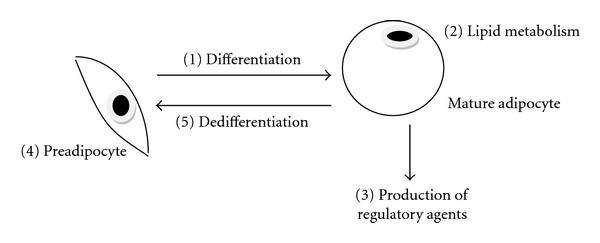
Strategic points in which the study of adipocytes will prove fruitful for obesity and metabolic-related problems. Traditional areas of concentrated research have focused on cell differentiation to form lipid-assimilating adipocytes (1), lipid metabolism under a variety of physiologies and nutrient loads (2), and (more recently) adipocyte production of local and systemic regulatory agents (3). However, new targets like deciphering the potential mechanisms of mature adipocyte dedifferentiation (5) to form proliferative-competent progeny cells like additional preadipocytes (4) are being carefully evaluated.

Figure 2 depicts five targets for manipulating adipocytes in order to regulate obesity or metabolic syndrome. Traditional research and clinical focus has been directed towards (1) and (2), the formation of lipid assimilating adipocytes from adipocyte precursors (preadipocytes). Moreover, as the stromal vascular cell fraction of any adipose depot may provide cells of the adipocyte lineage (preadipocytes) a majority of research has traditionally been placed on this cell population. Recent interest has been expanding to include mechanisms in which adipocytes play an active regulatory role in metabolism (3). To this end, data from recent studies suggest that fetal programming of mesodermal cells may play an important role in the accumulation of postnatal adipocytes [25]. Physiological relevant processes as simple as altering the diet of mothers might regulate adipocyte numbers in offspring. Alterations in adipocyte numbers have also been shown via diet manipulations at discernable time points postnatally (4). To be sure, the nutritional plane of mothers during pregnancy results in lower birth weights of babies so the ability to shift the overall cellular make-up during development is not absurd. Moreover, mature adipocyte numbers may not be as fixed as once thought. Adipocytes might be able to dedifferentiate to form additional proliferative-competent progeny cells (5) which might add adipocytes to specific adipose depots, thereby increasing the lipid load [12]. 

Collectively, knowledge gained in any of these areas will aid us in finding a possible regulatory point for combating energy flux-related dysfunctions. Similarly, the observations contained in Table 1 offer insight into physiological points/observations whereby research might provide knowledge regarding alleviation of adverse symptoms of both obesity and metabolic syndrome. As one example, since adipocytes are capable of synthesizing and releasing chemical regulators into the blood that might affect systemic metabolism/physiology, the identification of the release/effect mechanism might then point to a target for regulation (Figures 1 and 2). In light of this, a systemic remediation regimen might be developed, reducing the effects of adipocyte secretions.

## 5. Conclusion and Perspectives

Can adipose tissue become the major regulator of body composition? Why is it that one of the most benign/banal tissues, adipose tissue, has become an amazing source of stem cells and systemic regulatory agents and, therefore, controls of a variety of systemic and local physiological and pathological variables of growth, development, and metabolism? In the light of current research, this once thought innocuous tissue has emerged as a true organ system, and these are issues that have to be addressed in the near future to gain more understanding regarding obesity, metabolic syndromes, and adipocytes.

##  Conflict of Interests

The author declares that there is no conflict of interests.

## Figures and Tables

**Table 1:** Obesity, metabolic syndrome, adipogenesis, and angiogenesis.

Documented physiology	Reference
Moderate obesity is associated with adipocyte hypertrophy, whereas more severe obesity also involves adipocyte hypertrophy and hyperplasia. In obese pigs, hyperplasia is evident as clusters of small adipocytes.	[26]

Adipose tissue capillary endothelium changes markedly at the ultrastructural and structural level with adipocyte hypertrophy and even more so in obesity. Capillary lumen diameters are reduced considerably. These changes could interfere with the vascular remodeling necessary during adipocyte hypertrophy.	[27]

Angiogenic capacity was determined by quantifying capillary branch formation from human subcutaneous and visceral adipose tissue explants. subcutaneous explants had more capillary sprouting than visceral adipose tissue but this increased sprouting decreased with morbid obesity representing dysfunctional angiogenesis.	[28]

Angiogenesis associated with subcutaneous adipose tissue and visceral adipose tissue from the same obese patients was evaluated by laying adipose tissue on chick chorioallantoïc membranes. The angiogenic potency of adipose tissue was not depot or fat cell size dependent.	[29]

Pangenomic microarray analysis showed that inflammatory markers and acute phase reactants were overexpressed in obese compared to lean human subcutaneous adipose tissue. Modulation of the inflammatory pathways represents a new therapeutic target for the treatment of obesity and related complications. Genes associated with adipogenesis, per se, were not differentially expressed.	[30]

The development of methods for hypoxia detection in adipose tissue has indicated a hypoxia response in adipose tissue in obese animals. Adipose tissue hypoxia (ATH) may provide mechanisms for chronic inflammation, macrophage infiltration, and mitochondrial dysfunction among other features in adipose tissue in obesity. Adipose tissue blood flow associated with a failure in compensatory angiogenesis or vasodilatation may precipitate ATH. Translational studies in humans are necessary to provide conclusive evidence in support of the ATH concept.	[31]

The development and maintenance of the adipocyte extracellular matrix (ECM) is critical to maintain the function of the adipocyte. Hypoxia in obesity may destabilize the ECM resulting in a number of adverse conditions. Adipocyte hypertrophy may adversely influence the adipocyte ECM stability.	[32]
A 12 yr study of 11,326 respondents showed that overweight individuals with basal metabolic indices (BMI) ranging between 25 and 29.9 had 17% less relative risk of mortality than those with BMI below 18.5 or over 35.	[33]

Weight reduction by 5 to 10% of original weight reduces insulin resistance, blood glucose, blood lipids, and blood pressure, suggesting that some individuals adapt to the excess weight.	[4, 34]

Decline in plasma adiponectin or the rise in C-reactive protein, regardless of obesity, appears to be a better predictor of metabolic syndrome than obesity alone.	[35, 36]

The abatement of metabolic syndrome has been attempted through the use of the two isomers of congugated linoleic acid. The CLA trans10, cis12 isomer depresses differentiation of adipocytes by decreasing expression of peroxisome proliferator-activated receptor *γ* (PPAR*γ*). The dietary provision to rats of mixtures of CLA cis9, trans11 and CLA trans10, cis12, produced from industrial hydrogenation of vegetable oil, abated insulin resistance and normalized glucose tolerance. However, provision of only CLA trans10, cis12 induced insulin resistance in humans and mice, even though it was found to reduce adipogenesis. Normalization of glucose tolerance by the thiazolidinediones (TZD) is mediated by activation of lipogenesis through the PPAR*γ*-dependent transactivation of GLUT4 genes, which encourage glucose uptake and increase lipogenesis, rather than the antilipogenic mechanism suggested for CLA trans10, cis12. In addition, the provision of mixed isomers of CLA to rats, where the CLA cis9, trans11 is a recognized lipogenic factor, normalized insulin resistance, suggesting that limitations to lipogenesis may be involved in the manifestation of metabolic syndrome.	[37–42]

## References

[1] Trogdon JG, Finkelstein EA, Hylands T, Dellea PS, Kamal-Bahl SJ (2008). Indirect costs of obesity: a review of the current literature. *Obesity Reviews*.

[2] Popkin BM, Kim S, Rusev ER, Du S, Zizza C (2006). Measuring the full economic costs of diet, physical activity and obesity-related chronic diseases. *Obesity Reviews*.

[3] Fabricatore AN, Wadden TA (2006). Obesity. *Annual Review of Clinical Psychology*.

[4] Kiberstis PA (2005). A surfeit of suspects. *Science*.

[5] Flegal KM, Graubard BI, Williamson DF (2004). Methods of calculating deaths attributable to obesity. *American Journal of Epidemiology*.

[6] Flegal KM, Williamson DF, Pamuk ER, Rosenberg HM (2004). Estimating deaths attributable to obesity in the United States. *American Journal of Public Health*.

[7] Flegal KM (2006). Commentary: the epidemic of obesity—what’s in a name?. *International Journal of Epidemiology*.

[8] Flegal KM (2005). Epidemiologic aspects of overweight and obesity in the United States. *Physiology and Behavior*.

[9] Flegal KM, Carroll MD, Ogden CL, Johnson CL (2002). Prevalence and trends in obesity among US adults, 1999-2000. *Journal of the American Medical Association*.

[10] Cameron AJ, Shaw JE, Zimmet PZ (2004). The metabolic syndrome: prevalence in worldwide populations. *Endocrinology and Metabolism Clinics of North America*.

[11] MacLaren R, Cui W, Simard S, Cianflone K (2008). Influence of obesity and insulin sensitivity on insulin signaling genes in human omental and subcutaneous adipose tissue. *Journal of Lipid Research*.

[12] Hausman GJ, Dodson MV, Ajuwon K (2009). Board-invited review: the biology and regulation of preadipocytes and adipocytes in meat animals. *Journal of Animal Science*.

[13] Gavrilova O, Marcus-Samuels B, Graham D (2000). Surgical implantation of adipose tissue reverses diabetes in lipoatrophic mice. *The Journal of Clinical Investigation*.

[14] Seip M, Trygstad O (1996). Generalized lipodystrophy, congenital and acquired (lipoatrophy). *Acta Paediatrica, International Journal of Paediatrics, Supplement*.

[15] Lee K, Villena JA, Moon YS (2003). Inhibition of adipogenesis and development of glucose intolerance by soluble preadipocyte factor-1 (Pref-1). *The Journal of Clinical Investigation*.

[16] Reue K, Xu P, Wang XP, Slavin BG (2000). Adipose tissue deficiency, glucose intolerance, and increased atherosclerosis result from mutation in the mouse fatty liver dystrophy (fld) gene. *Journal of Lipid Research*.

[17] Moitra J, Mason MM, Olive M (1998). Life without white fat: a transgenic mouse. *Genes and Development*.

[18] Puri V, Czech MP (2008). Lipid droplets: FSP27 knockout enhances their sizzle. *The Journal of Clinical Investigation*.

[19] Nishino N, Tamori Y, Tateya S (2008). FSP27 contributes to efficient energy storage in murine white adipocytes by promoting the formation of unilocular lipid droplets. *The Journal of Clinical Investigation*.

[20] Hosooka T, Noguchi T, Kotani K (2008). Dok1 mediates high-fat diet-induced adipocyte hypertrophy and obesity through modulation of PPAR-*γ* phosphorylation. *Nature Medicine*.

[21] Unger RH (2002). Lipotoxic diseases. *Annual Review of Medicine*.

[22] Rhodes CJ (2005). Type 2 diabetes—a matter of *β*-cell life and death?. *Science*.

[23] Frayn KN (2001). Adipose tissue and the insulin resistance syndrome. *Proceedings of the Nutrition Society*.

[24] Frayn KN (2005). Obesity and metabolic disease: is adipose tissue the culprit?. *Proceedings of the Nutrition Society*.

[25] Du M, Yin J, Zhu MJ (2010). Cellular signaling pathways regulating the initial stage of adipogenesis and marbling of skeletal muscle. *Meat Science*.

[26] Hausman GJ, Martin RJ (1981). Subcutaneous adipose tissue development in Yorkshire (lean) and Ossabaw (obese) pigs. *Journal of Animal Science*.

[27] Hausman GJ, Richardson RL (1983). Cellular and vascular development in immature rat adipose tissue. *Journal of Lipid Research*.

[28] Gealekman O, Guseva N, Hartigan C (2011). Depot-specific differences and insufficient subcutaneous adipose tissue angiogenesis in human obesity. *Circulation*.

[29] Ledoux S, Queguiner I, Msika S (2008). Angiogenesis associated with visceral and subcutaneous adipose tissue in severe human obesity. *Diabetes*.

[30] Viguerie N, Poitou C, Cancello R, Stich V, Clément K, Langin D (2005). Transcriptomics applied to obesity and caloric restriction. *Biochimie*.

[31] Ye J (2009). Emerging role of adipose tissue hypoxia in obesity and insulin resistance. *International Journal of Obesity*.

[32] Mariman ECM, Wang P (2010). Adipocyte extracellular matrix composition, dynamics and role in obesity. *Cellular and Molecular Life Sciences*.

[33] Orpana HM, Berthelot JM, Kaplan MS, Feeny DH, McFarland B, Ross NA (2010). BMI and mortality: results from a national longitudinal study of canadian adults. *Obesity*.

[34] Cavan D, Cradock S (2004). Structured education programmes and Type 2 diabetes. *Diabetic Medicine, Supplement*.

[35] Pradhan AD, Manson JE, Rifai N, Buring JE, Ridker PM (2001). C-reactive protein, interleukin 6, and risk of developing type 2 diabetes mellitus. *Journal of the American Medical Association*.

[36] Zoccali C, Mallamaci F, Tripepi G (2002). Adiponectin, the most abundant adipocyte derived protein, is functionally related to metabolic risk factors and predicts cardiovascular outcomes in end stage renal disease. *Journal of the American Society of Nephrology*.

[37] He, ML, Mir PS, Okine E, Dodson MV (2003). Effect of culture media containing conjugated linoleic acid (CLA) cis (c) 9, trans (t) 11 and CLA t10, c12 on lipid accumulation in 3T3-L1 preadipocytes. *Journal of Animal Science*.

[38] McTernan PG, Harte AL, Anderson LA (2002). Insulin and rosiglitazone regulation of lipolysis and lipogenesis in human adipose tissue in vitro. *Diabetes*.

[39] Clément L, Poirier H, Niot I (2002). Dietary trans-10,cis-12 conjugated linoleic acid induces hyperinsulinemia and fatty liver in the mouse. *Journal of Lipid Research*.

[40] Risérus U, Arner P, Brismar K, Vessby B (2002). Treatment with dietary trans10cis12 conjugated linoleic acid causes isomer-specific insulin resistance in obese men with the metabolic syndrome. *Diabetes Care*.

[41] Belury MA, Moya-Camarena SY, Lu M, Shi L, Leesnitzer LM, Blanchard SG (2002). Conjugated linoleic acid is an activator and ligand for peroxisome proliferator-activated receptor-gamma (PPAR*γ*). *Nutrition Research*.

[42] Brown JM, McIntosh MK (2003). Conjugated linoleic acid in humans: regulation of adiposity and insulin sensitivity. *Journal of Nutrition*.

